# National estimates of kinship size and composition among adults with activity limitations in the United States

**DOI:** 10.4054/demres.2021.45.36

**Published:** 2021-11-10

**Authors:** Adriana M. Reyes, Robert F. Schoeni, Vicki A. Freedman

**Affiliations:** 1 Cornell University, School of Public Policy and Sociology, 2223 MVR Hall, Ithaca, NY 14853, USA.; 2 Institute for Social Research, University of Michigan, 426 Thompson Street, Ann Arbor, MI 48106, USA.

## Abstract

**BACKGROUND:**

The number of adults living with limitations in daily activities in the United States is large, and projected to increase. Families, which are becoming more complex, are critical to the wellbeing of this population.

**OBJECTIVE:**

We present national estimates of the size and composition of kin networks for adults with activity limitations.

**METHODS:**

We use the 2013 Panel Study of Income Dynamics to assess kin relationships of adults aged 40 and older with an activity limitation. We assess kin relations up and down one generation and horizontally, including spouses, adult children, parents, siblings, step-kin, parent-in-laws, children-in-law, and sibling-in-laws. We estimate kinship size and differences across race/ethnicity, education, and marital status. We also estimate the number of helpers.

**RESULTS:**

Adults with activity limitations have a substantial number of adult kin: 9.1 on average, while only 12% have fewer than four kin. Spouses and adult biological children, the most common caregivers, account for less than one-third of these kin. Kin networks are much larger among those who report their background as Hispanic rather than non-Hispanic white or Black, married rather than unmarried, and less-than-college rather than college-educated.

**CONCLUSIONS:**

Despite concerns about increasing family complexity, we find that 88% of individuals with a limitation have four or more family members, and as kin size increases the average number of kin helping increases from one to two.

**CONTRIBUTION:**

We provide estimates of kinship size and composition for adults with disabilities, assessing the number of kin, types of kin, and sociodemographic differences.

## 1. Introduction

Over 60 million adults have a disability, including sensory, cognitive, mobility, self-care, or independent living limitations, and rates of disability increase steeply with age (Okoro et al. 2018). For many people needing help with daily activities, family members are an important source of assistance. Kin provide assistance in a variety of ways, including help with household and self-care tasks or mobility needs, financial assistance, emotional and social support, direct health and medical care, advocacy and care coordination, surrogacy, and health information (National Academies of Sciences and Medicine 2016).

Despite the central role families play in helping adults cope with daily activities, analyses of how kinship size and composition vary for adults with activity limitations are lacking. Much of what is known focuses on older adults. For example, studies have found that older adults with a larger number of kin receive more assistance from their kin network in total (Checkovich and Stern 2002; Crimmins and Ingegneri 1990; Spitze and Logan 1990). Prior research also suggests that assistance received by older adults from each individual kin member falls as kinship size increases (Checkovich and Stern 2002; Engers and Stern 2002; Hiedemann and Stern 1999; Spitze and Logan 1991). Irrespective of size, spouses and biological children are more likely than other relatives to provide assistance to older adults with limitations (Wolff et al. 2018).

Given the central role kin play in providing help, we use nationally representative data to explore the size and composition of kin networks for adults living with activity limitations. Using data from the Panel Study of Income Dynamics (PSID), we assess how many family members are potentially available to be called upon to help with daily activities, what types of kin are available, and the magnitude of sociodemographic differences. We examine these patterns by socio-demographic characteristics and provide national estimates of the connection between kinship size and the number of kin actually providing help.

## 2. Methods

### 2.1 Data and sample

We use the 2013 wave of the PSID and the 2013 Rosters and Transfers supplement, a national longitudinal survey of the US population (Johnson et al. 2018). Given the increase in activity limitations starting at roughly age 40, we restrict the sample to the 7,758 adults (i.e., PSID reference persons and spouses/partners) aged 40 and older. We further restrict the sample to the 1,663 adults 40 and older with at least one limitation in an activity of daily living (ADL – Difficulty doing any of the following by oneself, without equipment: bathing or showering, dressing, getting in or out of bed or chair, eating, walking, getting outside, using the toilet) or instrumental activity of daily living (IADL – Difficulty doing any of the following by oneself: preparing meals, shopping for personal toilet items or medicine, managing own money, using the telephone, doing heavy housework, doing light housework).^3^ We then exclude 83 respondents who are missing or report ‘other’ race, for a final sample size of 1,580.

### 2.2 Measures

The Rosters and Transfers module includes questions about living biological parents (including whether each biological parent’s current spouse/partner is not the sample person’s biological parent, i.e., is a step-parent), living biological children 18 and older (including whether each such child has a spouse/partner), and the number of living biological full- and half-siblings (Schoeni et al. 2015). Because this information is also collected for spouses/partners, we can identify living stepchildren aged 18 and older, living parents-in-law, and living sisters/brothers-in-law from current marriages/partnerships.

We focus on both the number and type of kin relationship. Four broad categories of living adult kin are considered:

- Spouse: partner through marriage or romantic cohabitation of at least one year;
- Biological and adopted kin (henceforth ‘biological’): biological and adopted children aged 18 or older, biological and adoptive parents, and biological full- and half-siblings;
- Step-kin: stepchildren ages 18 or older and step-parents;
- In-laws (biological, adopted, or step): spouses of biological, adopted, and stepchildren; spouse’s biological, adoptive, and step-parents; spouse’s biological full- and half-siblings.

We also calculate the number of parent and adult-child helpers, where a helper is a couple or individual who the respondent reports as spending time providing assistance in the past year. Information on other helpers (e.g., siblings, non-relatives) is not collected. Time helping can be for everyday needs or for more episodic needs; no information is collected on the type of assistance. For adults who received assistance from a married or cohabiting parent or adult child, PSID data do not specify which member(s) of the couple provided the assistance. We therefore count the subset of parent and adult-child units who provided any time assistance in the past year and calculate the proportion of helpers as the number of units helping divided by the total number of parent and child units.

We examine kin size differences by age, gender, race/ethnicity, education, marital status, metropolitan status, and limitation severity. Age is included as a continuous measure in the models and is included categorically in describing kin size differences as those younger than 65, and 65 and older. Race/ethnicity is categorized as non-Hispanic white, non-Hispanic Black, and Hispanic. Years of completed education is categorized into 16 or more vs. less than 16 years. Marital status distinguishes those currently married or cohabitating for at least one year from non-partnered individuals. Metropolitan status distinguishes those living in metropolitan vs. non-metropolitan settings. We also assess the severity of limitations by examining differences between those who have fewer than two ADLs and those having two or more.

### 2.3 Analysis

We first estimate the proportion of adults with an activity limitation who have different kinship types, the mean number of kin of each type both conditional and not conditional on having that kin type, and the percentage of all kin by relationship type. We then examine the composition of kin networks by the number of kin. Next, we estimate age differences in the size and composition of kinship types and use Poisson regression^4^ to estimate age- and sex-adjusted kinship size and differences across gender, race/ethnicity, education, marital status, metropolitan status, and limitation severity. Finally, using linear regression and controlling for age, gender, race/ethnicity, education, marital status, and number of limitations, we estimate among those receiving help the relationship between the number of parent and child units and two outcomes: the number and the proportion of helpers. We display predicted values from the models. All analyses are weighted using the PSID cross-sectional weight adjusted for immigration since 1997 and adjusted to account for the PSID oversample of Black parents in 1997 (Freedman and Schoeni 2016). We conduct additional sensitivity tests that examine differences by limitation severity and duration to test for differences between ongoing and temporary limitations.

## 3. Results

**Figure 1:**
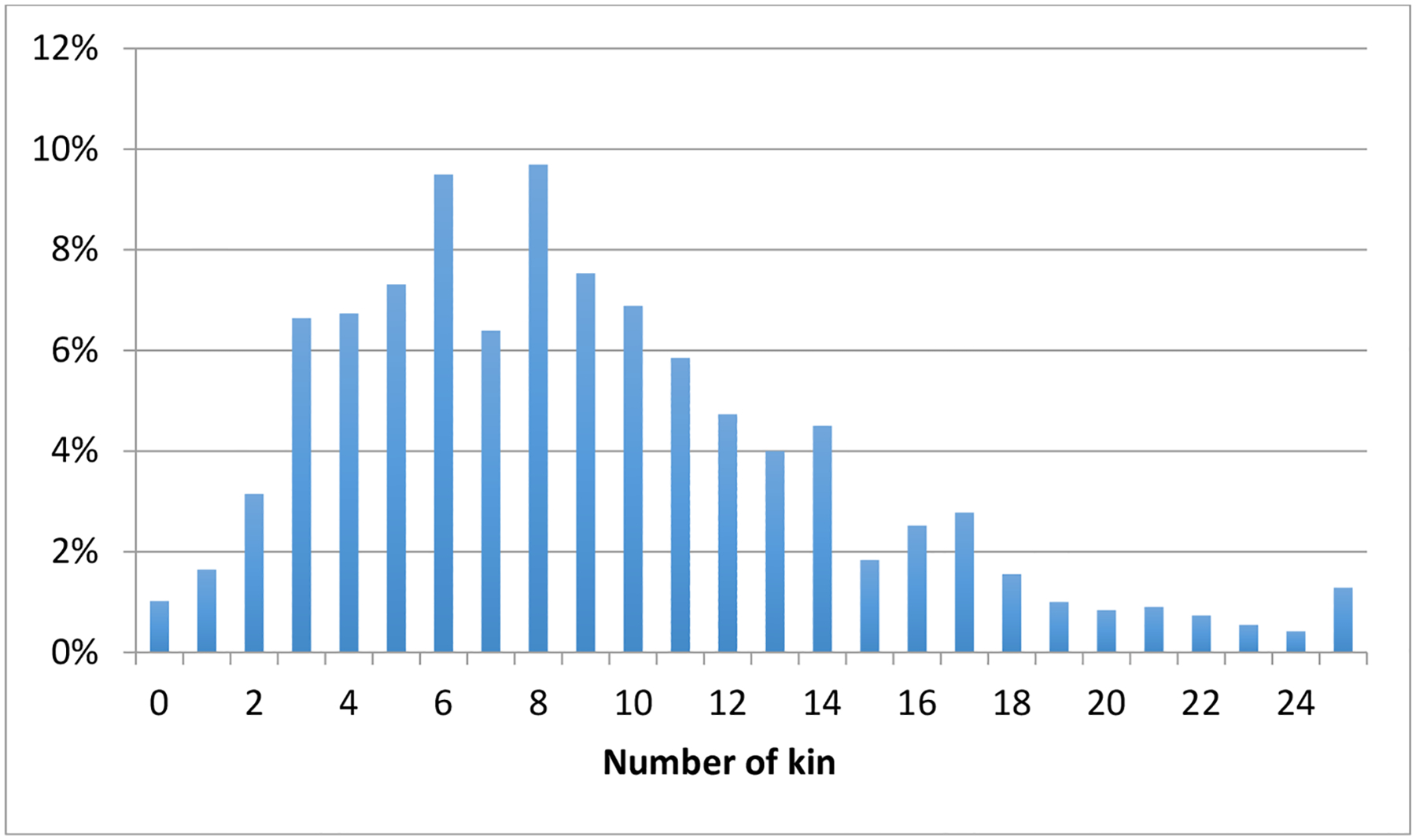
Number and percentage of kin in sample of adults 40+ with activity limitation *Source*: PSID, 2013, N = 1,580.

Among adults aged 40 and older living with an activity limitation, 98.8% have at least one kin member (Table 1, column 1). Just 12.4% have fewer than four kin, while 22.9% have more than twelve (Figure 1). On average, adults with limitations have 9.1 family members (Table 1, column 3); 63.8% of family members are spouses or biological relatives and the remaining 36.2% are step-kin or in-laws (Table 1, column 4).

Most adults with limitations (8 out of 10) have adult biological children and siblings, 62.6% have at least one child-in-law, about half have a spouse, 47% have a sibling-in-law, and 30.9% have a biological parent (Table 1, column 3). Reflecting declines in fertility more generally, on average this population has more siblings (3.1) than children (2.7) (Table 1, column 2).

Among kin, siblings are most common (28.6%), followed by biological children (25.3%), siblings-in-law (15.4%), and children-in-law (12.1%); spouses account for just 5.5% of all kin (Table 1, column 4). Stepchildren account for 11.5% of all adult children, and step-parents represent 20% of all parents (Table 1, column 4).

**Figure 2:**
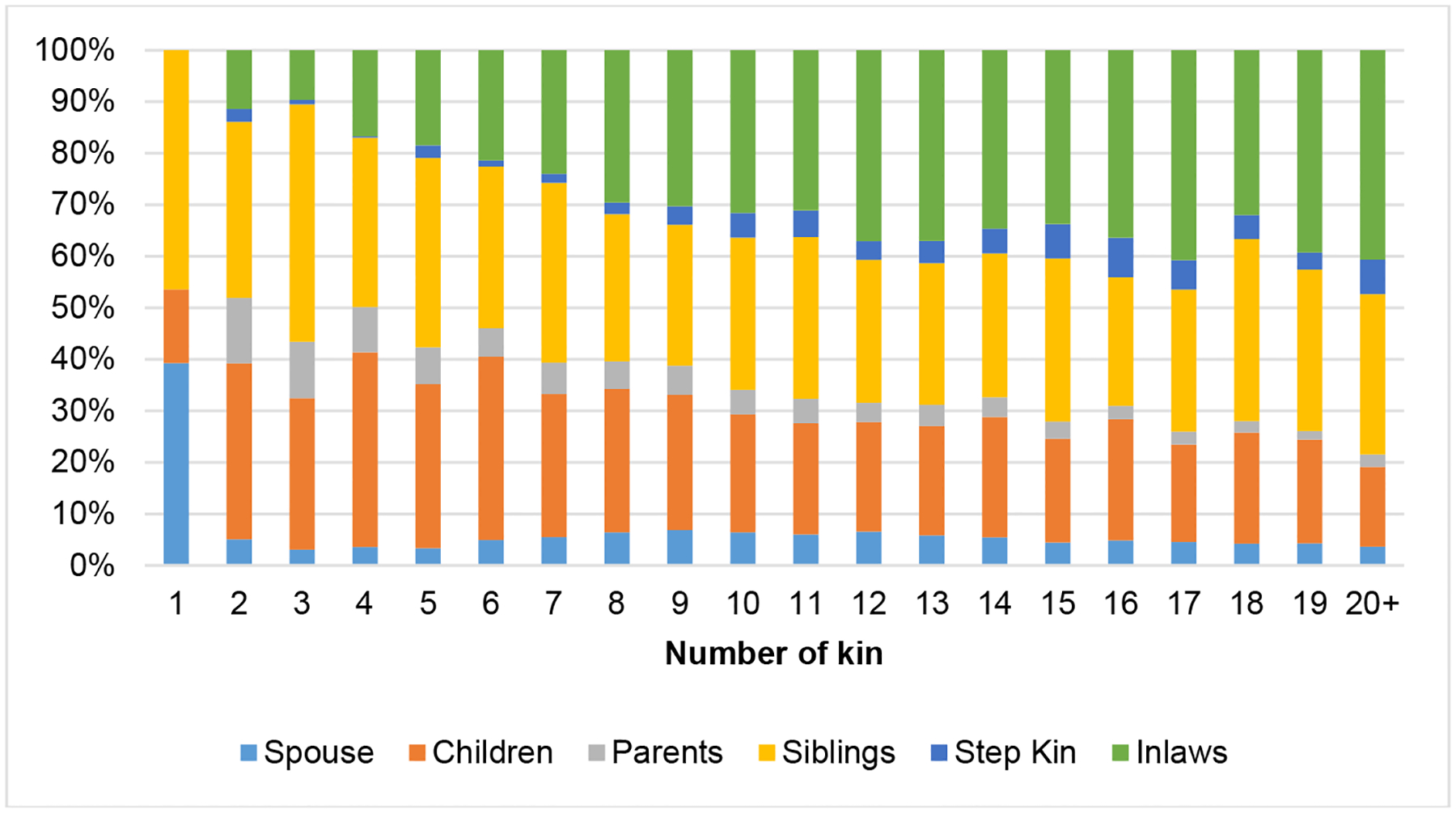
Composition of kin among adults with activity limitation *Source*: PSID, 2013, N = 1,580.

As the number of kin increases, the composition and diversity of kin types changes (Figure 2). For those with only one living family member, almost half have only a living sibling and about 39.3% have only a spouse. At the average kin size (about 9), spouses are a much smaller share of the kin network whereas children, siblings, and in-laws comprise the majority of family members. With increasing kinship size, in-laws represent a greater share, siblings represent a substantial and consistent share, and step-kin and parents represent a consistently small share.

As shown in Table 2, younger persons also have larger kinship size than persons over age 65, largely driven by having about twice as many living parents and siblings. Men on average have about 1 more relative than women (9.7 v. 8.8), partially because men have more in-laws than women. This may also reflect survivorship among widowed women.

The bottom panel of Table 2 presents age- and sex-adjusted estimates from the Poisson regression. We find Hispanic and Black adults with limitations have larger kinship networks (13.4 and 9.3, respectively) compared to non-Hispanic white adults (8.3). The larger kinship size for Black adults is driven by having considerably more biological children and siblings than non-Hispanic whites. Hispanic adults with limitations have notably more biological children, siblings, and in-laws compared to non-Hispanic whites.

Those with a college education have fewer family members than those without a college degree (8.1 v. 9.3). Educational differences in total kin size are observed because those with a college education have fewer biological children and siblings. However, those with a college degree are more likely to be married. Adults with an activity limitation who are married have much larger kinship networks than those who are not married (11.7 v. 6.6), largely because they have more biological children and in-laws in their network. We observe no differences in kinship size or composition by metropolitan status. Adults with two or more ADLs have one less kin member than those with fewer ADLs (8.5 v. 9.5); being less likely to have a spouse and having fewer in-laws accounts for more than half of the difference. In sensitivity analyses, we found no difference in kinship size between those with temporary and ongoing limitations (not shown).

**Figure 3:**
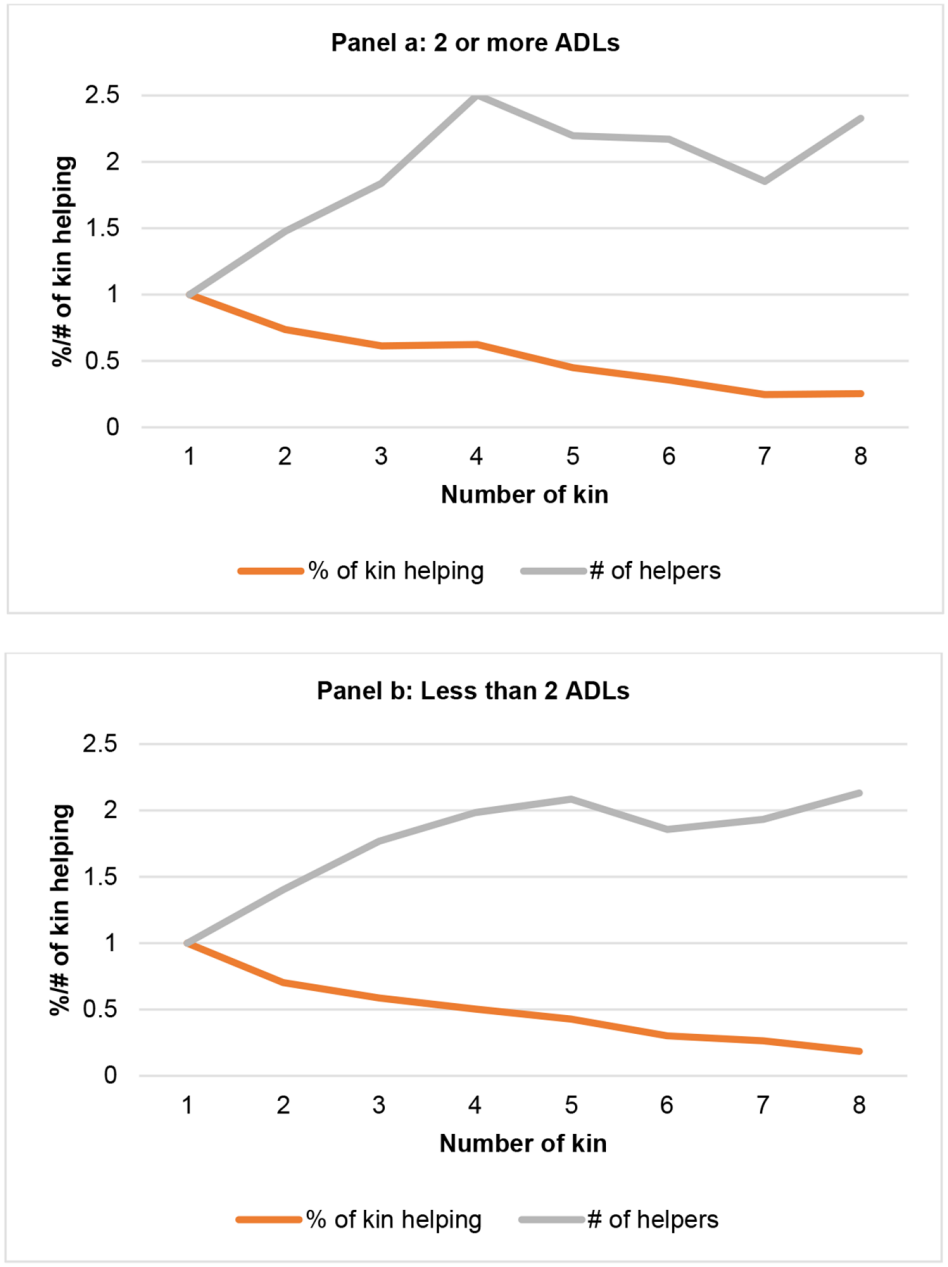
Number and proportion of parent and adult-child helping units by kin size and number of ADLs *Note*: Predictions based on linear regression models estimating the number and proportion of parent and adult child helping units among those reporting any help, controlling for age, gender, race/ethnicity, marital status, education, and number of limitations. *Source*: PSID, 2013, N = 648.

About half the sample reports receiving help at any one time; and among those receiving help the average number of helpers is slightly larger among those with two or more (vs. less than two) ADLs (Figure 3). For both groups, the number of helpers increases as kin size increases but only up to about 4 or 5 parent and child units, at which point the average number of helpers levels off at about 2 helping units. Thus, as kin size increases beyond 4, individuals with limitations are activating a smaller proportion of their kin network. Additional sensitivity analyses comparing those with temporary vs. ongoing limitations and differences by age are presented in the Appendix (Table A-1).

## 4. Discussion

We note three important contributions of this analysis of the kin size and composition of adults aged 40 and older living with an activity limitation. First, our results provide new estimates of kin size among adults with an activity limitation across groups and find extremely few with no or limited available kin. We find that only 1% of older adults with an activity limitation have no living kin, and 4% have no living parents or children. These estimates are similar to estimates from previous research on kinlessness (Margolis and Verdery 2017); however, having no kin may be especially detrimental for those with activity limitations because not only do they not have a family caregiver but they also lack the emotional support having family may provide. Groups that have higher chances of developing limitations – Black, Hispanic, and less-educated adults – have larger kin networks, and particularly more biological family members, than non-Hispanic whites and those with a college degree, potentially offsetting their increased likelihood of needing assistance.

Second, we provide estimates of the types of kin that comprise kin networks, providing important insights for thinking about family complexity and the provision of care. Siblings and in-laws represent a substantial share of kin networks, but very few currently provide intensive help (Schulz et al. 2016). One barrier to sibling and in-law care is lack of their inclusion in family care policies and programs. More research is needed to understand when and how these and other family members may be activated to care for those in need and to better understand the relationship between kinship size and composition and caregiving. Additionally, as kin size becomes larger, step-kin and family complexity become more prevalent. Stepchildren and step-parents account for 12% and 20% of all children and all parents, respectively, and these shares will likely rise because family complexity is more common in recent cohorts, many of which have yet to reach ages where physical limitations are more prevalent (Agree 2018). Existing evidence suggests that step ties are weaker than biological ties (Wiemers et al. 2019); thus, the increase in family size from step-kin may have a limited effect on caregiving, especially when biological kin are available to fulfill needs. More studies are needed to determine how increasing kin size from step ties and family complexity are related to caregiving.

Third, we show novel estimates that the relationship between number of kin and the number of helpers has a ceiling effect. Among adults with an activity limitation who report receiving help, the number of helpers increases with kin size up to only about four family units, maxing out at about two helpers. This means that as kin size increases a smaller proportion of the kin network is activated. Studies that suggest recent family trends will result in fewer caregivers do not take into account that most adults with limitations have on average two caregivers, no matter how large (or small) their kin network. This finding helps contextualize concerns about decreasing family size and suggests that the ceiling effect regarding the number of helpers may lessen the implications for caregiving. Research looking at the number of caregivers by level of assistance needed also finds an average of about two caregivers (Freedman and Spillman 2014). More research is needed to understand whether this pattern arises because needs are being met, because strong ties that facilitate helping behavior exist with a small proportion of kin, or because only a limited set of kin are available to help.

Coresidence and proximity of kin are also important aspects of kin networks for understanding caregiving dynamics, as previous research has suggested that coresidence and proximity enable caregiving (Litwak and Kulis 1987). Unfortunately, we are unable to assess coresidence or proximity of all kin types. The study is also limited by not being able to account for extended kin such as grandchildren, aunts, uncles, and kin from former marriages. Regarding step-parents, we do not know if they were a parental figure for the focal person, though only 6.5% report having a step-parent. Additionally, the 2013 PSID sample is not representative of more recent cohorts of immigrants (Duffy and Sastry 2012), though we do attempt to address this limitation by using weights that adjust for immigrant underrepresentation. Finally, these results may not be generalizable outside of the US context, as familial expectations and norms regarding caregiving vary across the world.

## 5. Conclusions

Using nationally representative data to estimate kinship size among those with an activity limitation, we find that almost all have at least one family member, and complexity of kin types increases with kin size. We find that on average only two kin provide help, regardless of kin size, suggesting increased family complexity may not drastically alter current care arrangements. Future research should seek to understand if this is due to family availability or because needs are being met. This research note provides a national profile of family size and composition for adults with an activity limitation and finds most have large kin networks.

## Figures and Tables

**Table 1:** Kinship size by type of kin among adults ages 40 and older with an activity limitation (N = 1,580)

		Average number of kin among:		
	% with given kin type [1]	those with kin of given type [2]	all persons [3]	% of kin who are each type* [4]
**Spouse**	50.0	1.0	0.5	5.5
**Biological Kin**				
Children 18+	83.6	2.7	2.3	25.3
Parents	30.9	1.3	0.4	4.4
Siblings	84.7	3.1	2.6	28.6
***Step-kin***				
Stepchildren 18+	14.7	2.1	0.3	3.3
Step-parent	5.3	1.2	0.1	1.1
***In-laws (bio or step)***				
Spouses of children	62.6	1.9	1.1	12.1
Spouses of stepchildren	9.6	1.7	0.2	2.2
Spouse’s parent	19.9	1.4	0.3	3.3
Spouse’s step-parent	2.6	1.2	0.03	0.3
Spouse’s siblings	47.0	3.2	1.4	15.4
***Total kin size***	**98.9**	**9.2**	**9.1**	

*Notes*: PSID, 2013. Weighted using adjusted survey weight.

* Refers to the percentage of total kin that is comprised of each kin type (e.g., # children 18+/# of kin = 2.3/9.1 = 25.3)).

**Table 2:** Kinship size by key demographics among adults 40 and older with an activity limitation

	Total	Spouse		Biological children		Biological parents		Siblings		Step kin		Inlaws	
	Size	Size	% Type	Size	% Type	Size	% Type	Size	% Type	Size	% Type	Size	% Type
**Age**													
40–65	9.8	0.5	5.1	2	20.4	0.7	7.1	3.4	34.7	0.4	4.1	3.1	31.6
65+	8.5	0.5	5.9	3.1	36.5	0.05	0.6	1.9	22.4	0.2	3.5	3	35.3
P-value	(0.000)	(0.067)		(0.000)		(0.000)		(0.000)		(0.001)		(0.574)	
Age adjusted^†^													
**Gender**													
Men (ref)	9.7	0.6	6.2	2.5	25.8	0.2	2.1	2.6	26.8	0.4	4.1	3.4	35.1
Women	8.8	0.4	4.5	2.5	28.4	0.2	2.3	2.5	28.4	0.3	3.4	2.8	31.8
P-value	(0.012)	(0.000)		(0.737)		(0.880)		(0.328)		(0.001)		(0.001)	
Age and sex adjusted^†^													
**Race/Ethnicity**													
White (ref)	8.3	0.5	6.0	2.3	27.7	0.2	2.4	2.1	25.3	0.3	3.6	2.8	33.7
Black	9.3	0.3	3.2	2.8	30.1	0.1	1.1	3.6	38.7	0.2	2.2	2.1	22.6
P-value	(0.026)	(0.000)		(0.002)		(0.012)		(0.000)		(0.147)		(0.006)	
Hispanic	13.4	0.6	4.5	3.4	25.4	0.2	1.5	4.2	31.3	0.4	3.0	4.8	35.8
P-value	(0.000)	(0.164)		(0.000)		(0.326)		(0.000)		(0.243)		(0.000)	
**Education**													
Less College (ref)	9.3	0.5	5.4	2.6	28.0	0.2	2.2	2.6	28.0	0.3	3.2	3	32.3
College	8.1	0.6	7.4	2.1	25.9	0.3	3.7	2	24.7	0.3	3.7	2.8	34.6
P-value	(0.001)	(0.006)		(0.000)		(0.000)		(0.000)		(0.734)		(0.155)	
**Marital status**													
Single (ref)	6.6	0	0.0	2.2	33.3	0.2	3.0	2.5	37.9	0.1	1.5	1.3	19.7
Married	11.7	1	8.5	2.9	24.8	0.2	1.7	2.6	22.2	0.5	4.3	4.7	40.2
P-value	(0.000)			(0.000)		(0.113)		(0.595)		(0.000)		(0.000)	
**Metro status**													
Non-Metro (ref)	9.2	0.5	5.4	2.6	28.3	0.2	2.2	2.4	26.1	0.3	3.3	3.2	34.8
Metro	9.1	0.5	5.5	2.5	27.5	0.2	2.2	2.6	28.6	0.3	3.3	2.9	31.9
P-value	(0.662)	(0.846)		(0.361)		(0.811)		(0.269)		(0.486)		(0.239)	
**Number of ADLs**													
0–1 ADL (ref)	9.5	0.5	5.3	2.5	26.3	0.2	2.1	2.7	28.4	0.3	3.2	3.2	33.7
2+ ADLs	8.5	0.4	4.7	2.5	29.4	0.2	2.4	2.3	27.1	0.3	3.5	2.7	31.8
P-value	(0.003)	(0.016)		(0.894)		(0.001)		(0.019)		(0.662)		(0.005)	

Notes:

† Estimates from Poisson regression models controlling for age or age and gender.

P-values in parentheses reflect group differences in comparison to the noted reference category (ref) for each characteristic in Poisson regression models.

*Source*: PSID, 2013, N = 1,580.

## Footnotes

3 A small number of respondents rely on a family member to provide a proxy response (6%). Those with severe limitations are more likely to have a proxy response; however, excluding these respondents does not substantively alter findings.

4 We find little evidence of overdispersion and models estimated using negative binomial regression produce similar estimates and standard errors.

## Appendix

**Table A-1:** Predicted number of helpers by kin size across sub-groups

Number of parent and adult-child units	Ongoing limitation	Temporary limitation	Age 40–64	Age 65 and older
1	1	1	1	1
2	1.5	1.2	1.3	1.5
3	1.8	1.4	1.6	1.9
4	2.4	1.8	2.2	2.4
5	2.0	1.6	2.2	2.0
6	2.1	1.2	1.6	2.3
7	2.0	1.7	2.2	1.7
8	2.6	2.2	2.8	2.1

*Note*: Predictions based on linear regression models estimating the number of parent and adult-child units helping among those reporting any help, controlling for age, gender, race/ethnicity, marital status, education, and number of limitations.

*Source*: PSID, 2013, N = 648.
